# Impact of COVID-19 on the mobility patterns: An investigation of taxi trips in Chicago

**DOI:** 10.1371/journal.pone.0267436

**Published:** 2022-05-05

**Authors:** Satyam Mukherjee, Tarun Jain

**Affiliations:** 1 Shiv Nadar University, Greater Noida, India; 2 Indian Institute of Management Bangalore, Bangalore, India; Universidade Estadual de Maringa, BRAZIL

## Abstract

The COVID-19 outbreak has impacted urban transportation mobility throughout the world. In this paper, we investigate the impact of COVID-19 on the urban mobility network’s structural characteristics. We contribute to the literature by discussing how various community areas in the city traffic network are impacted by the pandemic. We analyze a large dataset on urban mobility from the city of Chicago and derive various insights. Our analysis of the mobility network structure is important because a better understanding of such networks can help control the spread of the disease by reducing interactions among individuals. We find that the pandemic significantly impacted the structure of the mobility network of taxis in Chicago. Our study reveals some important pointers for policymakers that could potentially aid in developing urban transportation policies during the pandemic.

## 1 Introduction

The COVID-19 outbreak has drastically led to various operational issues in society [1]. Recent studies on the COVID-19 pandemic have shown how the global crisis has impacted global supply chains [2–4], bed capacity for patients in hospitals [5, 6], mobile service operations [7], and omnichannel retailers [8]. Lockdown measures aimed at mitigating the spread of the virus, have significantly affected human mobility [9–11]. Since the mobility of infected people from one location to another may lead to the spread of infection, the transportation networks are changing during the current crisis [12]. Moreover, it is important to understand the human transportation networks as this may help policymakers easily control the spreading of the disease by limiting contact among individuals [13–15]. During the COVID-19 crisis, the public-transient ridership has fallen from 70 percent to 90 percent [16]. This is because the spread of the disease has halted everyday life, and therefore, urban transportation activities [17].

Understanding daily mobility pattern changes due to the pandemic helps policymakers analyze how various communities respond to social distancing measures. Further, this helps various decision-makers deploy appropriate resources in certain city localities so that social distancing measures are properly implemented in high-density traffic zones. To facilitate better operations management of transportation networks in smart cities, large-scale datasets on daily travel usage and end-user feedback need to be collected. Due to recent advances in creating, transferring, storing, and analyzing digital data, a high volume of data on the daily mobility in smart cities data is generated and captured at multiple sources [18]. Large public sector organizations in Chicago are providing comprehensive open data to the public [19]. In our analysis, we utilize such large-scale datasets to explore structural changes in transportation networks arising from taxi trips in Chicago.

In Chicago city, approximately 7000 cabs operate. During the pre-COVID 19 periods, the Chicago city taxi transportation network was responsible for an average of 32,917 trips per day. However, during the ongoing crisis, this number reduced to an average of approximately 1553 trips per day. In this study, we explore whether the weighted networks of origin-destination (OD) community areas in Chicago endured structural changes during the COVID-19 pandemic. We provide insights on the impact of the pandemic on the taxicab network of Chicago.

### 1.1 Research question and contributions

In this study, we utilize the complex social networks analysis approach to analyze large-scale taxi trip data collected in Chicago, USA and explore the structure of taxi travel mobility networks. To understand the impact of COVID-19 on the city mobility network, we analyze the dynamic traffic network from January 2020 to October 2020. Previous research on smart city operations has utilized the taxi-trip data to investigate the traffic flows in the cities [20, 21]. We further contribute to previous literature on pandemic management and smart city operations by analyzing the impact of the pandemic outbreak on traffic flows. This study is important because various structural characteristics of city mobility networks like weighted betweenness centrality, weighted degree centrality, and weighted local clustering coefficient are important factors that determine the high demand zones, highly probable travel zones, and the traffic clusters in the city mobility networks [22, 23]. Further explorations of the above structural aspects of mobility networks in pre-COVID-19 and post-COVID-19 traffic networks provide new insights for transportation and city management under pandemic outbreaks [24, 25].

In our study, we construct the daily mobility networks with various community areas in Chicago city as the nodes of the network and a trip between two community areas represents the edge of the network. After constructing the traffic networks, we address the following question: *How are mobility patterns impacted in different parts of the city due to the outbreak of COVID-19*? *Are there any structural differences in the traffic network mobility patterns under the ongoing pandemic compared to pre-COVID-19 times*?

Our analysis reveals that initially, due to the pandemic outbreak, there is a rapid decrease in the overall city traffic mobility, followed by some recovery in mobility. Finally, we observe the stability in overall mobility. However, during the period when mobility is stable, overall mobility is lower than pre-pandemic levels. Next, we find a heterogeneous impact of disease spread on mobility in various community areas (or zone in the city). For example, we observe that the community areas characterized by high economic activities faced a sharper decline in the overall mobility during the onset of the pandemic compared to the regions associated with low economic activities.

We also estimate betweenness centrality and local clustering coefficient for community area (or city zone) in the traffic networks using social network analysis tools. The betweenness centrality of a community area in a network is defined as the fraction of the number of shortest paths that pass through a community area over the total number of shortest paths in the network. Further, the local clustering coefficient that quantifies the local density of a community area can be estimated as the fraction of the number of links with the neighbors of a community area to the number of possible connections in the network. Our analysis reveals that at the onset of the pandemic, the weighted betweenness centrality increased and peaked; after that, it steadily decreased. Finally, our research shows that the local clustering coefficient increased and attained a peak value after the pandemic outbreak, indicating the increase in the short-distance mobility to nearby areas. After that, we observe the local clustering coefficient has decreased to a stable value.

The rest of the paper is organized as follows: Section 2 positions our paper in the existing literature. Section 3 discusses the materials and methods where we describe the taxi-trips dataset and the network construction strategy. We present the analysis and discuss the results in Section 4. Finally, the conclusions and the suggestions for policymakers based on the results stated in the paper are discussed in Section 5 of the paper.

## 2 Literature review

In this research, we contribute to two streams of research. The first stream of research deals with the operational issues in smart city planning. The second stream of research relevant to our work deals with decision making under the pandemic crisis. Next, we review each of the above streams.

### 2.1 Smart city operations

Our work has interesting implications for planners of the smart city while designing the logistic network during a healthcare crisis. We refer readers to the review of recent papers on challenges and opportunities in smart city operations [26–28]. Prior research on air transportation visualized the impact of weather disruption on the world wide air traffic network [29]. Research on the robustness of air traffic networks under natural hazard events studied the loss and recovery of critical network functionality (measured by traffic volume and node degree) in the US National Airspace System Airport network [30]. Unlike them, we analyze the impact of the pandemic event on the taxi traffic flow.

There are papers in the literature that present various approaches to model the road travel demand. A recent study investigated the impact of various factors such as income, petrol price, number of petrol stations, number of rail stations, and number of bus stops on the vehicle miles traveled [31]. Earlier research estimating pedestrian demand within a train station uses various indicators such as train timetable data, ridership data, link flow data, and Origin-Destination flow data [32]. Further research involving a multiclass speed-density relationship in pedestrian movement utilizes real-life data to test the performance of the approach [33]. Again, research involving a dynamic discrete choice-based demand model replicates timing decisions, trip length, and trip duration for daily travel activity [34]. Finally, researchers have evaluated multi-modal travel itineraries by considering the reliability of the chosen transportation services [35]. Unlike the above papers, we adopt a social network approach to analyze the impact of the pandemic outbreak on traffic flow.

Finally, there is a set of papers that utilize various techniques from social network analysis to study the structural aspects of smart city mobility networks [36, 37]. The closest papers in this stream study the impact of traffic patterns in smart city operations. For example, an empirical analysis of street networks from 97 cities found that the distribution of betweenness centrality is a good discriminator for comparing congestion patterns and their evolution across various cities [38]. Recent research on traffic networks has used network science to study mobility patterns in a city using trip data [20, 21, 39]. Like us, they analyze the large-scale dataset from New York Taxi and Limousine Commission, and trip data in Wuhan city and Shanghai city. However, unlike them, we study the impact of the outbreak of COVID-19 on the Chicago city mobility network.

### 2.2 Pandemic management

We refer readers to the review of literature on the impact of epidemic outbreaks on supply chains [1]. Recently, various papers studied the impact of the COVID-19 outbreak on various operational issues in society [24, 25, 40]. Researchers analyzing the impact of COVID-19 on the credit risk of firms through linkages to global supply chains found that during different phases of COVID-19, the credit risk of the firms is impacted by disruption and resumption of supply chains [2]. A simulation study predicting the short-term and long-term impacts of the COVID-19 outbreak on global supply chains observed that the timing of the closing and opening of the facilities is a relatively more important factor as compared to the speed of epidemic propagation that determines the impact of the epidemic on the supply chains [3]. Another analysis involving computer simulation (based on a mid-size hospital) on managing the elective surgery backlogs after COVID-19 disruption found that if the hospitals can double the capacity and if the number of waiting for patients triples (as compared to pre-COVID-19 levels), in the best case it will take around five months to return the elective surgery rates to the pre-COVID-19 levels [6]. While studying the fixed-cost-subsidy, operations-cost-subsidy, and safety-technology-support schemes by the government to support mobile service operations (near customer’s home) during the pandemic outbreak, researchers observed that operations-cost subsidy generates higher consumer surplus as compared to fixed-cost subsidy [7]. An empirical investigation of the customer transactions dataset reveals that online channel sales increased due to the acquisition of new customers and switching of customers who previously purchased physical stores [8].

An empirical analysis of the impact of COVID-19 on music streaming consumption revealed that the COVID-19 outbreak reduced music consumption by 12.5 percent [41]. Utilizing process control charts to detect the surge in google searches of COVID-19 symptom keywords, authors have shown that the 3-sigma rule from statistical process control is suitable for the early-warning notification policy for the pandemic’s notification [42]. A dynamic transmission model aimed at helping the government and hospitals suggests that aggressive testing to detect and isolate infected persons could help in ending the outbreak [43]. A recent work using data from various countries evaluates various forecasting methods to estimate the growth of COVID-19 [44]. In contrast, we analyze the impact of COVID-19 on road traffic patterns and suggest some implications for policymakers.

Thomas et al. (2021) utilize survey data to analyze the change in travel attitudes due to COVID-19 [45]. They find that travel attitude was negatively affected due to the pandemic; however, they observe some recovery after travel restrictions were removed. Unlike them, we analyze the impact of COVID-19 on the traffic network using a large scale dataset from the city of Chicago. Overall, it remains an open question how the transportation networks are impacted and how such networks evolve before and after the enforcement of lockdowns across the globe, which we address in this paper.

## 3 Materials and methods

In this section, we explain the dataset considered in the study, the construction of traffic mobility networks of Chicago community areas at a daily aggregate level, followed by the three widely used network properties—weighted degree (or strength), weighted betweenness centrality, and weighted local clustering coefficient of a node.

### 3.1 Data and traffic network construction

In this subsection, first, we discuss the taxi-trips data in Chicago. Next, we portray the travel patterns using OD community areas in Chicago. We implemented a network approach to better understand how the travel patterns are affected by the COVID-19 pandemic.

#### 3.1.1 Data

**Fig 1:**
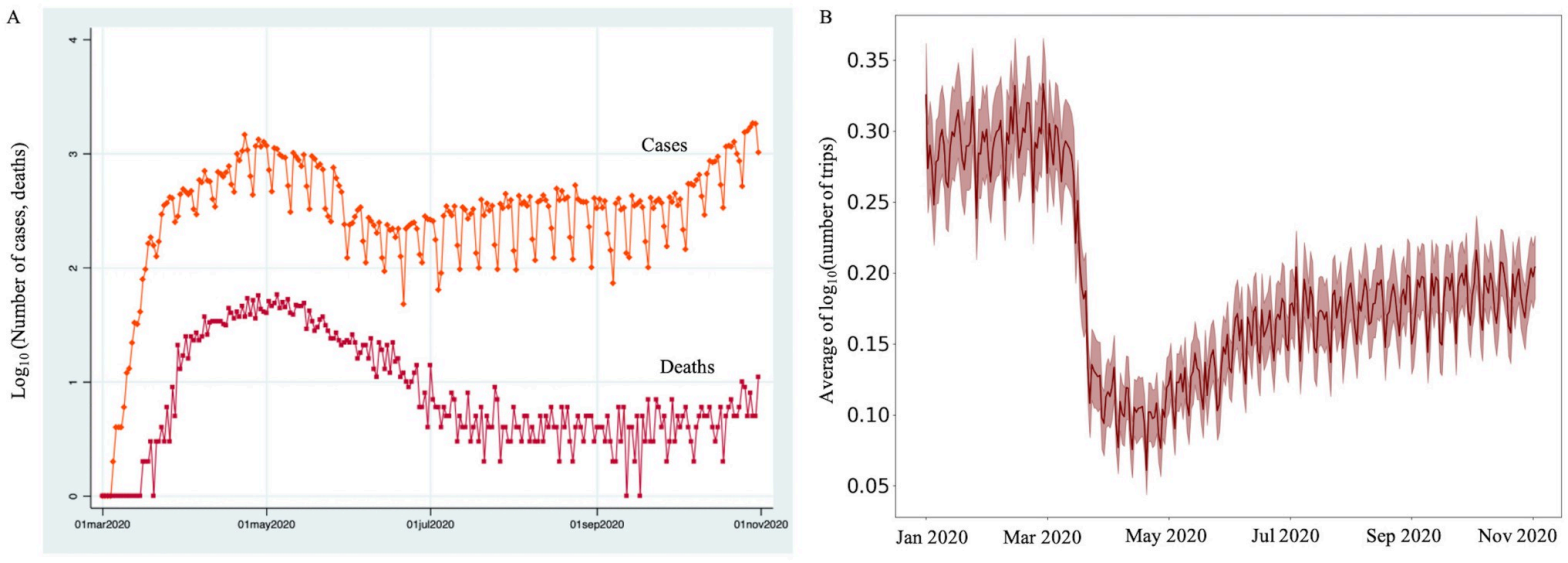
(A) We note a rise in COVID-19 cases and deaths from March 2020. (B) During the same period, the average number of taxi trips (log10 transformed) significantly drops.

Several important factors prompted us to select Chicago taxi trips as an appropriate case study for examining the effect of COVID-19 on smart city operations. In the state of Illinois, one of the earliest cases was reported in Chicago when a person traveled to the city from Wuhan, China in January 2020. Chicago is one of the largest cities in the United States of America (US), with an estimated population of 2,693,976 as of the 2019 US Census. By March 2020, Chicago became one of the major hotspots of COVID-19 cases in the US [46]. Fig 1A shows the growth of COVID-19 cases and deaths in Chicago between March and October 2020.

In the Supporting Information to the paper, we show the map of Chicago along with details of various community areas (or zones). The study area includes all the 77 community areas in Chicago. The taxi-trip dataset of Chicago comprises 3,597,297 trips over 10 months between January 01 and October 31, 2020. The trips also include the information of origin and destination community areas, duration, the distance of the trips, time of pick-up and drop-off, centroid location of the origin, and destinations. In addition to the trip data, we also gather the administrative boundaries of Chicago in the form of a shapefile. We use a Python script to derive the information from the shapefile (.shp format). Further, we access the data on COVID-19 cases, and deaths for Chicago [47].

#### 3.1.2 Network construction

Network-based studies provide a more nuanced understanding of travel patterns and spatial interactions between geographical locations [48]. Taxi trips in urban cities can be envisaged as a densely connected network of various zones or community areas. Building upon the extant literature on transportation networks, we construct networks of community areas in Chicago [20, 22, 30, 39, 49]. The pairs of nodes representing OD locations are connected if at least one taxi trip exists between two nodes. We construct a weighted and undirected network of taxi trips per day, where nodes represent Chicago community areas, and edges embody the total number of trips between OD locations. The weight of an edge is aggregated over the number of taxi trips per day.

#### 3.1.3 Traffic mobility network

The mobility of Chicago taxis between pick-ups and drop-offs in community areas can be portrayed as a network of origin and destination networks (OD networks). We leveraged a large dataset of Chicago taxi trips between January 01 and October 31, 2020. The taxi-trip management system in Chicago collects the geospatial information of the community areas and the date and time of trips. The geospatial information about origin-destination pairs (OD pairs) provides a platform to investigate human mobility patterns [50]. Aggregating the taxi trips between community areas in Chicago, we construct weighted networks of OD pairs, which approximate the human mobility behavior in big cities. Any change in human mobility patterns is reflected in the OD locations of the taxis. For example, when new cases of COVID-19 were reported in different parts of the US, the effect was reflected in the trip patterns of taxis in big cities [51]. Next, we elaborate on various metrics studied in the paper that capture the dynamics of the traffic mobility network.

#### 3.1.4 Degree centrality of traffic community area

One of the first basic indicators of network metrics is the degree of centrality of a node, defined as the number of direct neighbors of the node. Considering the number of taxi trips between two nodes in a network, a more significant network metric is obtained. Extending the concept of degree centrality to its weighted version, we define the strength (*s*_*i*_) of a node *i* as follows:

$$s_{i}=\sum_{j=1}^{N}A_{i}{}_{j}w_{i}{}_{j},$$

where *w*_*ij*_ is the weight of a link estimated as the number of taxi trips between two nodes *i* and *j*, and *A*_*ij*_ is the adjacency matrix of the network, whose elements take the value of 1 if two nodes *i* and *j* are connected: and 0 otherwise. In the traffic flow network, the high degree centrality area corresponds to the community area with a higher volume of starting and ending trips. Typically, the community area with a high degree centrality corresponds to areas with shopping complexes, office spaces, city centers, train stations, airports, and other commercial areas. Overall, the high degree centrality community areas are typically high travel demand zones.

#### 3.1.5 Betweenness centrality of traffic community area

While degree centrality (unweighted networks) or strength (weighted networks) of a node provides an intuitive measure of the central nodes of a network, prior studies have demonstrated that nodes with lower values of degree centrality or strength act as bridges in a network. A commonly used network metric that measures the centrality of nodes is betweenness centrality. Relying on the identification of shortest paths in a network, the betweenness centrality of a node is estimated as the fraction of shortest paths that pass through a node over the total number of shortest paths in the network. The weighted betweenness centrality considers the shortest paths as well the weight of the links. Formally, the weighted betweenness centrality ($BC_{i}^{w}$) of node *i* is defined as follows:

$$BC_{i}^{w}=\sum_{q,k\neq i;\sigma_{qk}\neq 0}\frac{\sigma_{qki}^{w}}{\sigma_{qk}^{w}},$$

As mentioned earlier, the number of taxi trips between two community areas quantifies the weight of a link. Additionally, we also normalize the metric by dividing by $\frac{1}{\left(N-1\right)\left(N-2\right)}$, where *N* is the network size. In the context of a mobility network, the community areas with high betweenness centrality correspond to areas with a traffic flow volume relatively higher than other areas [52]. Typically, the community area with a high betweenness centrality corresponds to the areas with highways, airports, and regions in the city’s central part [53].

#### 3.1.6 Local clustering coefficient of traffic community area

The local clustering coefficient quantifies the local density of a node in a network. It is measured as the fraction of the number of links to the number of possible links between the neighbors of a node. In the current study, we are interested in incorporating the number of taxi trips and estimating the weighted local clustering coefficient of a community area in Chicago. The weighted local clustering coefficient combines the topological properties of the network with the weight distribution, where the weight of a link is measured as the number of taxi trips along with the link each day. The weighted local clustering coefficient ($C_{i}^{w}$) of a community area *i* is defined as follows:

$$C_{i}^{w}=\frac{1}{s_{i}(k_{i}-1)}\sum_{jh}\frac{\left(w_{ij}+w_{jh}\right)}{2}A_{ij}A_{jh}A_{hi},$$

where *s*_*i*_ is the strength of a community area, *k*_*i*_ is the degree of *i*, *w*_*ij*_ is the number of taxi trips between two community areas *i* and *j*, and *A*_*ij*_ is the adjacency matrix taking the value of 1 if there exists at least one taxi trip between two community areas *i* and *j*; 0 otherwise. The weighted local clustering coefficient quantifies the local cohesiveness of the community area by taking into account the frequency of taxi trips. Higher values of $C_{i}^{w}$ would imply interconnected triplets are more likely to form between links with a higher number of taxi trips. Overall, the clustering coefficient quantifies the probability that two traffic areas directly connected to a third traffic area are also directly connected [22].

## 4 Results

Fig 1B illustrates the average number of trips (log10 scale) per day between January 01, 2020, and October 31, 2020. We observe three distinct regions in Fig 1B. Before March 11, the number of trips (log10 scale) fluctuates around the mean value of 0.3 (s.d. = 0.472), since the situation had not grown to a pandemic. Over the next two weeks, from March 11, 2020, the average number of taxi trips steadily declined as the number of COVID-19 infected cases started mounting. About the same period through the third week of March 2020, auto retail sales in the US suffered a 22 percent decline [54]. The steady decline in the average number of trips coincides with the travel ban from Europe (March 11), closure of schools, restaurants, and businesses in impacted areas in the US (March 16), “Shelter in Place” orders for Illinois (March 18), and Governor’s stay-at-home executive order (March 26) [19]. Furthermore, from March 26, 2020, the average number of trips keeps fluctuating around a mean value of 0.1 (s.d. = 0.220), almost following the same pattern before March 11. It is interesting to note that the average number of taxi trips initially dropped and, on average, got stabilized from March 26. However, the number of COVID-19 cases and deaths due to the pandemic kept increasing after March 26, attaining a peak around May 01, 2020 (see Fig 1A).

There could be several reasons for taxi trips not shutting down completely in Chicago despite the stay-at-home order and increasing number of COVID-19 cases. One possible factor could be the efforts of the Department of Business Affairs and Consumer Protection in providing subsidies for the taxi industry. The concerned authorities ensured taxi operations remain functional, particularly for the Americans with Disabilities Act (ADA) and paratransit services for essential trips [51]. Another possibility is commuters and taxi drivers are localized within nearby neighborhoods. Finally, front-line workers rely on transportation, while residents must have access to essential services. Accounting for all the above factors, it is reasonably justified that the taxi trips never hit an all-time low. While the number of trips provides a coarse estimate of the travel demands, it cannot comprehensively capture the interactions between community areas in the city. Therefore, we explore the network-level characteristics and elucidate the structure of commuting behavior during the pandemic.

**Fig 2:**
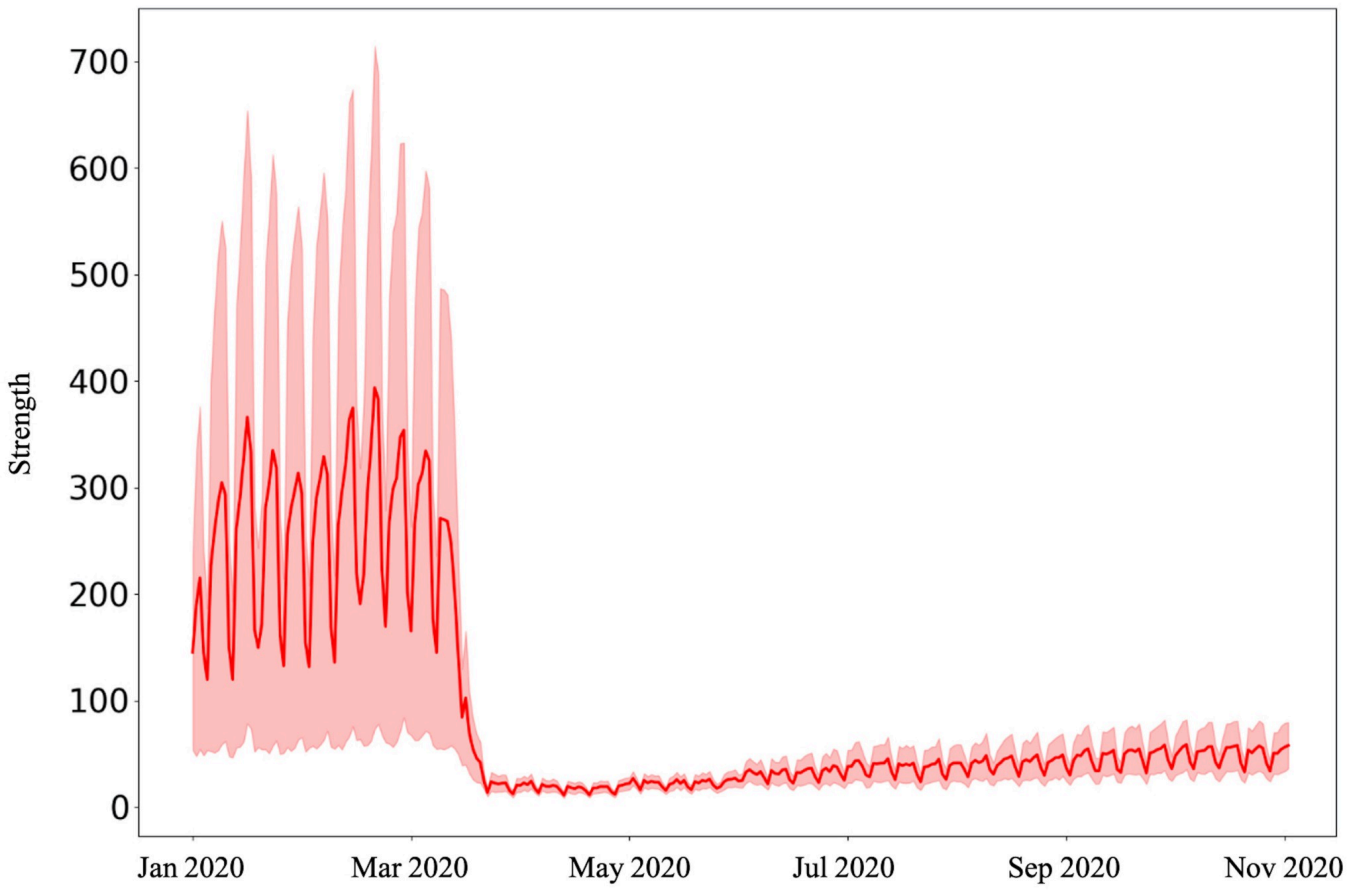
The average strength of community areas declines steadily from 256.65 (on March 11, 2020) (s.d. = 937.90) and stabilizes around an average of 18.31 (s.d = 22.42) (from March 26, 2020).

**Fig 3:**
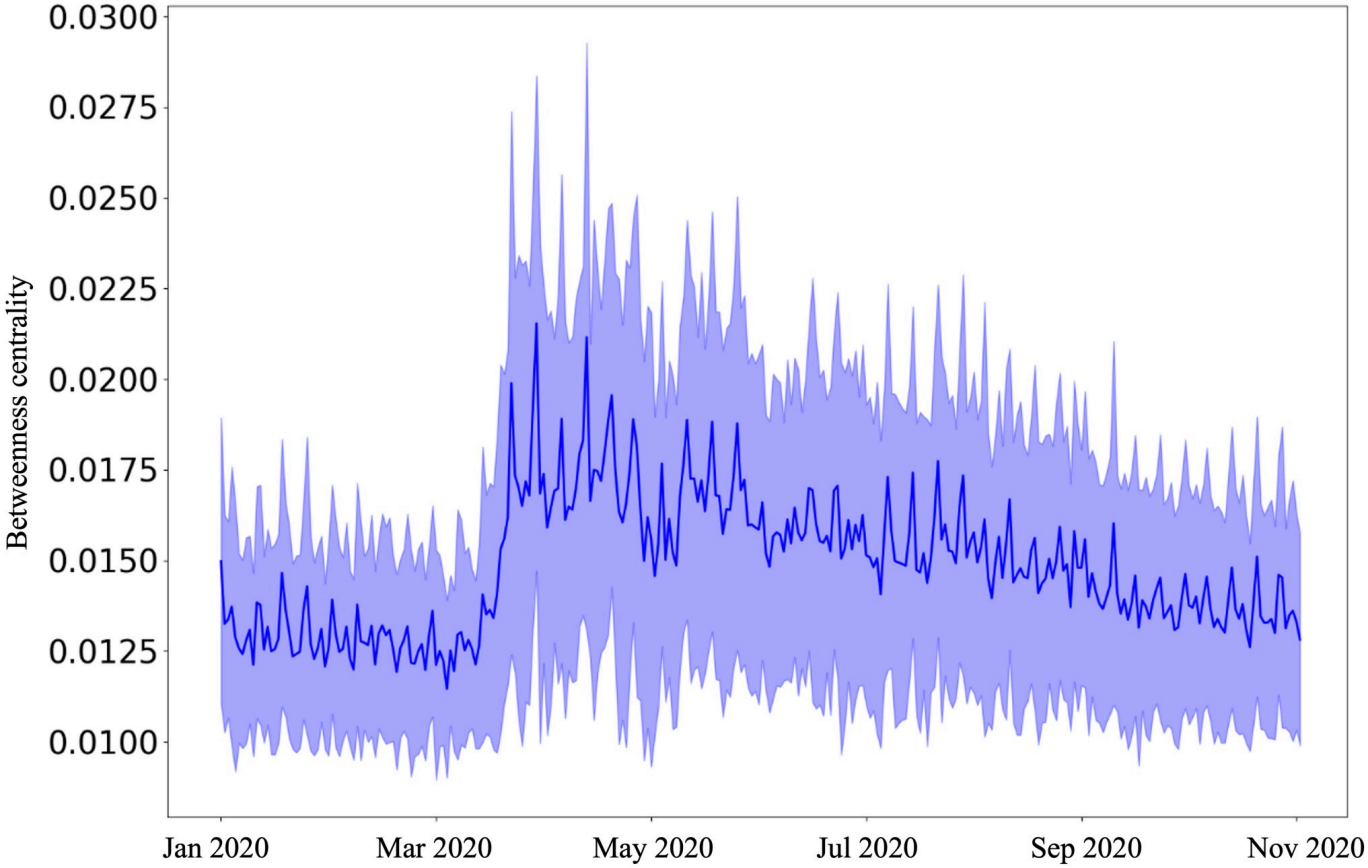
The weighted betweenness centrality increases and attains a peak about May 01, 2020, and steadily decreases ever since.

**Fig 4:**
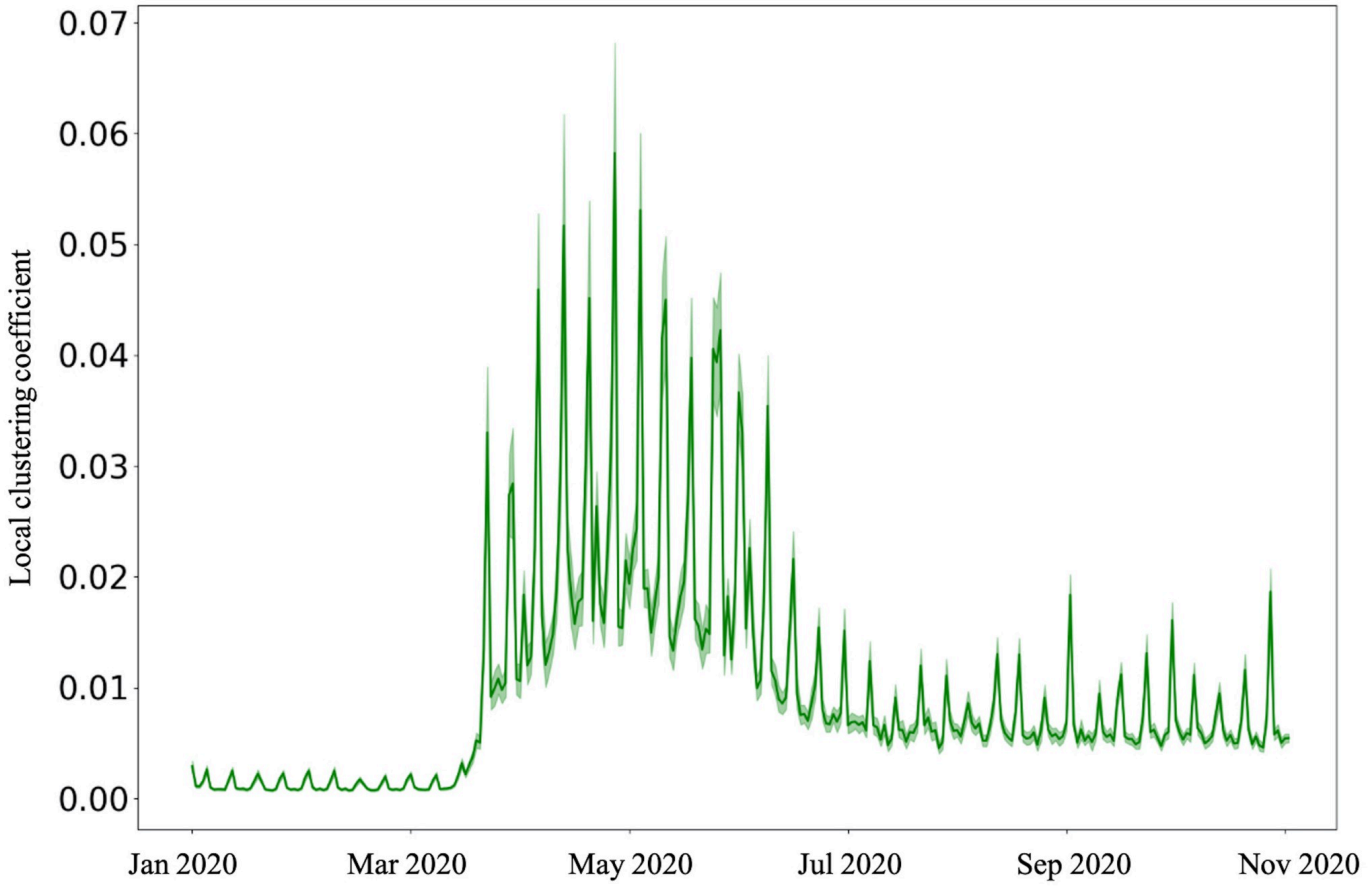
The weighted clustering coefficient increases with fluctuations attain a peak by about May 01, 2020, and decreases to a stable value from July 2020.

In Figs 2–4, we present the average of the metrics of the weighted and undirected network of OD pairs and compare the measures across the five most important community areas in Chicago. We first examine across the network-level characteristics. It is not surprising that the average strength of community areas declines steadily from 256.65 (on March 11, 2020) (s.d. = 937.90) and stabilizes around an average of 18.31 (s.d = 22.42) (from March 26, 2020) (see Fig 2). The large variation in the strength of zones before and after the stay-at-home order could be attributed to the overall drop in travel demands in different zones of Chicago.

Unpacking further, we examine the varied effects of the pandemic on weighted betweenness centrality and weighted clustering coefficient. It is interesting to note that while the strength of nodes decreases and attains a steady value, both weighted betweenness centrality and weighted local clustering increase from March 11, 2020, suggesting a surge in a greater number of alternate paths between community areas. Both the metrics attain their peak values on May 01, 2020. While the weighted betweenness centrality steadily decreases (Fig 3), the weighted local clustering fluctuates around a mean of 0.007 (s.d. = 0.004) from July 2020 (Fig 4). Both these metrics are extremely high during the weekends after the stay-at-home orders.

**Fig 5:**
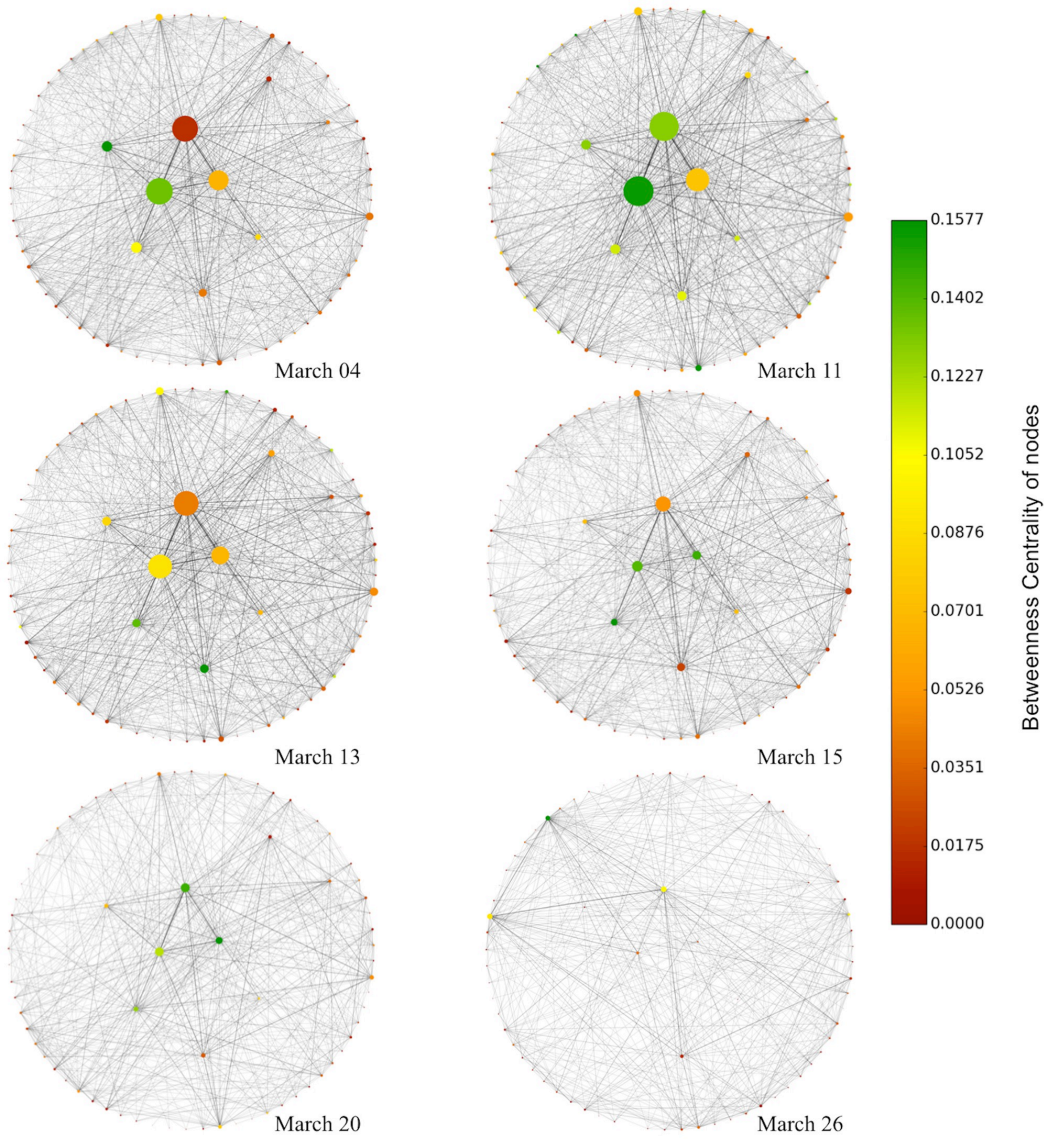
The color of the nodes (Chicago community areas) is proportional to the weighted betweenness centrality. The size of the nodes is proportional to the strength of the nodes (community areas) (log10 transformed).

The time-series data in Figs 2–4 provides the magnitude of information of the network metrics but neglects the interaction between the community areas in Chicago. In Fig 5, we provide the network visualization for six dates—March 04, March 11, March 13, March 15, March 20, and March 26, thus covering the duration where COVID-19 outbreak effects were reflected in the average number of trips (Fig 1). The nodes are colored as per weighted betweenness centrality, and the size of a node is proportional to its strength. We find that due to the decrease in the number of taxi trips in Chicago, the total number of edges in the networks has decreased significantly with time. Even though the node size has decreased, the color spectrum does not shift towards the red scale, rather the tendency is to shift towards the green scale. A possible explanation for this non-intuitive observation of low strength and high weighted betweenness centrality could be that taxi trips related to essential services and front-line duties are more relaxed in choosing multiple routes between OD locations. Next, we investigate the spatial distribution of the three network metrics for the chosen dates in March 2020.

**Fig 6:**
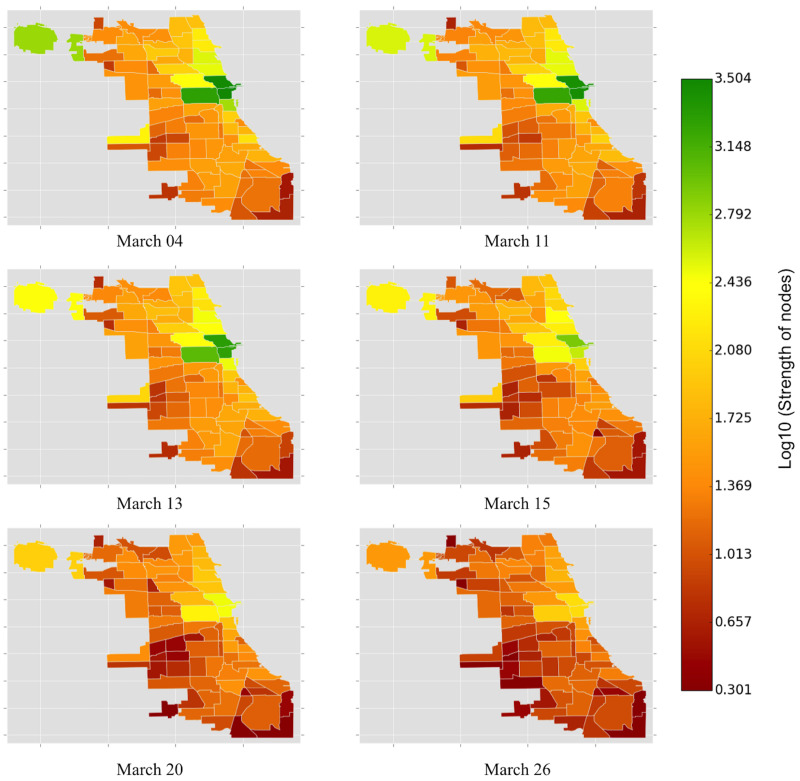
The color of the Chicago community areas is proportional to the strength of the nodes (community areas) (log10 transformed). On March 04, we observed higher values of node strength in Near North Side, West Town, Near West Side, Loop, and Chicago O’Hare. The node strength of Chicago O’Hare keeps decreasing from March 11, 2020, suggesting lesser movement of domestic and international travelers. The color of the zones neighboring the Loop shifts towards the red spectrum from March 15, 2020. Interestingly, the southern neighborhoods of Chicago do not witness any significant change in node strength.

**Fig 7:**
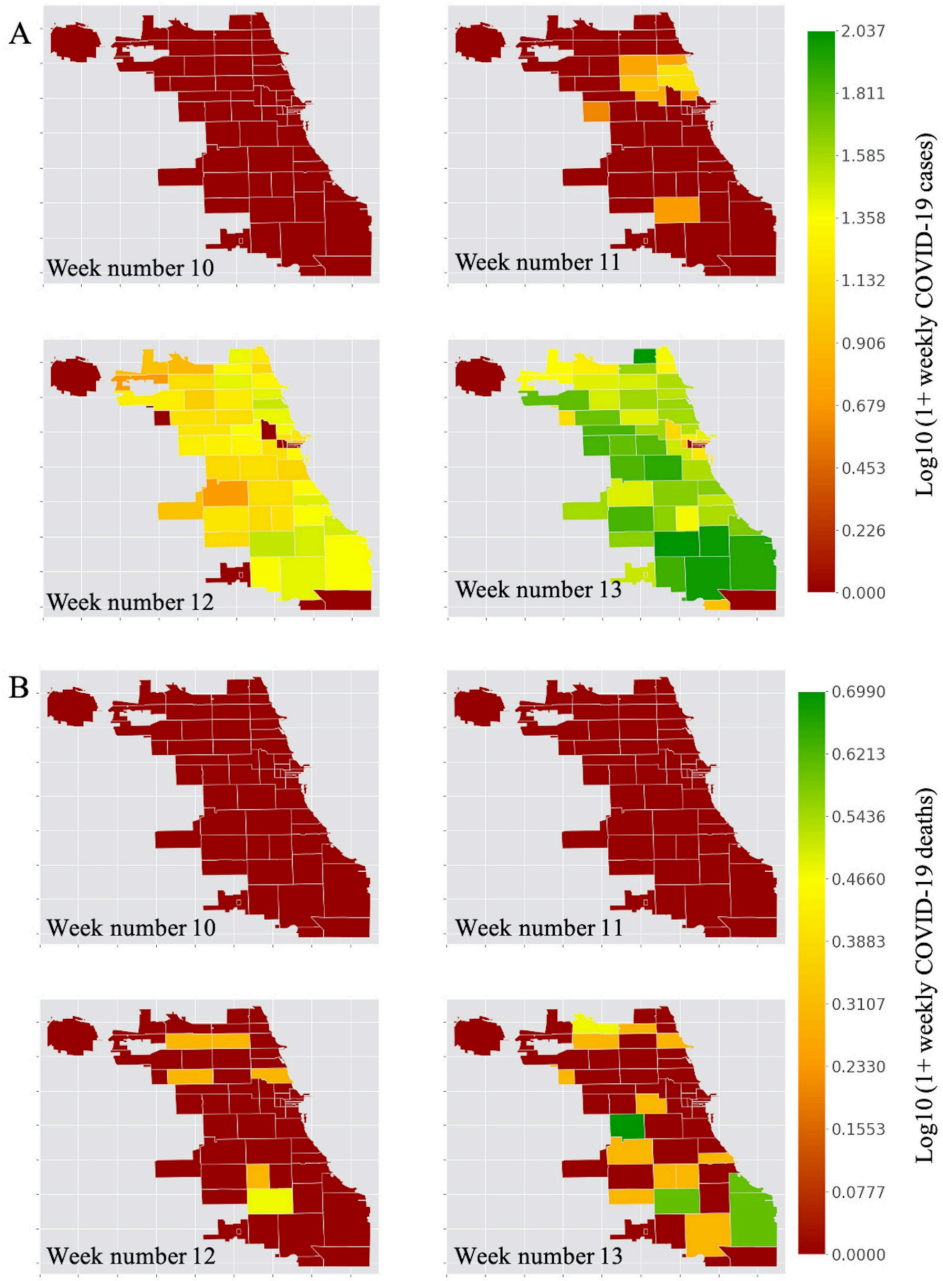
The color of the Chicago zip code is proportional to the weekly COVID-19 cases (log10 transformed). (A) The number of COVID-19 cases spreads almost uniformly across all the taxi zones by the 13^th^ week of 2020. (B) The deaths related to COVID-19 appear to have greatly affected the southern taxi zones of Chicago as compared to the other zones.

In Fig 6, we show the strength of nodes (community areas) for the chosen dates in March 2020. Further, in Fig 7, we present the number of COVID-19 cases (Fig 7A) and the number of deaths (Fig 7B) across various community areas in the city. The strength of nodes provides a reckonable representation of the level of interactions between origins and destinations in Chicago. The color spectrum of strength of community areas (Fig 6) could be interpreted as the range of activities in different locations in Chicago. On March 04, we observed higher values of node strength in Near North Side (community code 8), West Town (community code 24), Near West Side (community code 28), Loop (community code 32), and Chicago O’Hare (community code 76). Since the Loop is the commercial hub of Chicago, containing several retail establishments, restaurants, and commercial workplaces, higher values of node strength in and around the Loop is posteriori justified. Similar arguments hold for Chicago O’Hare, which primarily occupies the O’Hare International airport.

During the onset of COVID-19 in Chicago and the subsequent lockdown announcement, the node strength in O’Hare attains a lower value on March 11, 2020, indicating an overall avoidance of domestic and international travel. However, the node strength in and around the Loop area remains almost the same as of March 04, 2020. The decline of node strength for every community area in Chicago between March 13 and March 20 could be attributed to the emergence of the pandemic in Chicago (Fig 7). We also note that the decline of node strength in the Loop and its neighborhood covers the entire spectrum from green to red, indicating a sharp decline in activities in the Loop. In contrast, the activities in the Southern and Southwestern community areas in Chicago remain relatively stable around the red spectrum even during the onset of the pandemic. This can be explained by the sparse travel patterns within these community areas.

**Fig 8:**
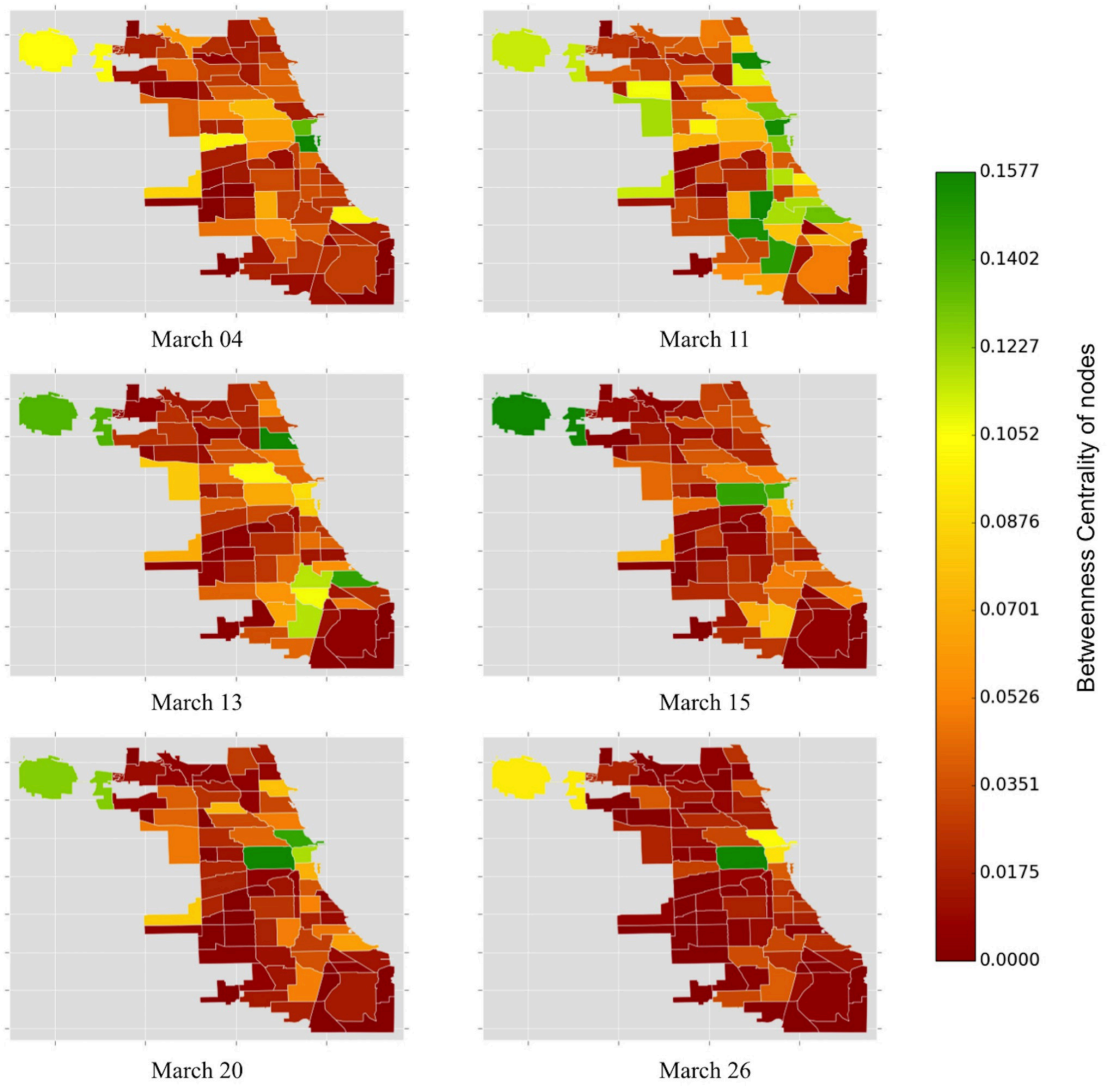
The color of the Chicago community areas is proportional to the weighted betweenness centrality of the nodes (community areas). Zones with a higher number of COVID-19 cases and deaths appear to have relatively lower values of weighted betweenness centrality. On March 13, 2020, we observe a surge in the weighted betweenness centrality for Lake View, O’Hare, South Shore, Greater Grand Crossing, Chatham, and Roseland. During the weekend (March 15), O’Hare experiences a further surge in weighted betweenness centrality. Again, on March 20 and 26, 2020 we note that O’Hare, Loop, and Near West Side have high values of weighted betweenness centrality.

Fig 8 depicting the weighted betweenness centrality of community areas reveals many interesting insights. Higher values of weighted betweenness centrality imply greater traffic loads in community areas. In other words, taxis are impeded in prominent community areas of the Loop, and the Near South Side (community area code 33). On March 04, 2020, we observe high values of weighted betweenness centrality in the Loop and Near South Side. Comparing with Fig 6, we note that these community areas have high values of strength as well, indicating there are few options for alternate paths in and around the neighborhood of Loop. Interesting spatial patterns of the community areas emerge in the following weeks. On March 11, community areas in the North (Uptown) and Southwestern regions (Roseland, Englewood, and Ashburn) of Chicago have high values of weighted betweenness centrality. As suggested in Fig 6, these regions are associated with lower values of strength, indicating that taxi drivers find alternating paths with a decline in the number of trips.

Comparing Figs 7 and 8, we deduce that zones with a higher number of COVID-19 cases and deaths have low values of weighted betweenness centrality. On Friday (March 13), we observe a surge in the weighted betweenness centrality for Lake View (community code 6), O’Hare, South Shore (community code 43), Greater Grand Crossing (community code 69), Chatham (community code 44), and Roseland (community code 49). During the weekend (March 15), O’Hare experiences a further surge in weighted betweenness centrality. Interestingly, on both these dates, the centrality values are relatively low for the adjacent neighborhoods in the Loop, perhaps indicating a combined effect of the weekend and the pandemic has discouraged commuters from traveling to popular locations on Near South Side and Near North Side.

We also observe a shift in the color spectrum toward the red color with intermittent green spectrum in certain community areas in the 3^rd^ week of March 2020. Focusing on March 20 and 26, we note that O’Hare, Loop, and Near West Side have high values of weighted betweenness centrality. At the same time, the node strength of these community areas is low (see Fig 6), suggesting that taxi commuting is restricted to only the major hubs of the city. With commuters avoiding commutes from the northern and southern suburbs of Chicago, the shortest paths are restricted only among the adjacent neighbors of the commercial cores of Chicago.

**Fig 9:**
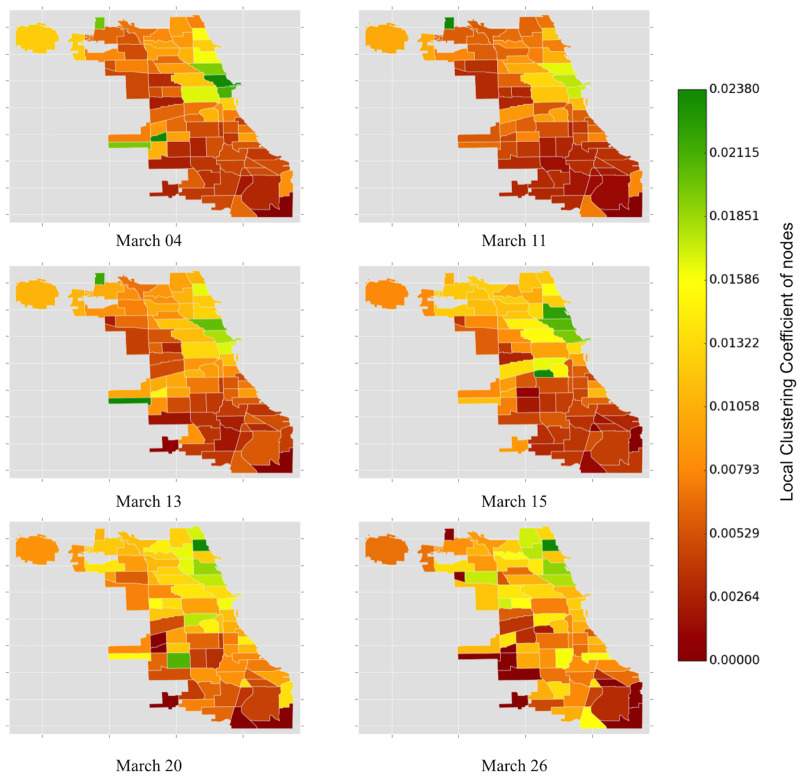
The color of the Chicago community areas is proportional to the weighted local clustering coefficient of the nodes (community areas). The heat map of the weighted local clustering coefficient complements the observation in Fig 4. The color spectrum of the taxi zones appears to be shifting towards green during the onset of the pandemic.

Fig 9 shows the variation of weighted local clustering for 77 community areas in Chicago over the same dates of observation. The weighted clustering coefficient quantifies the extent to which community areas tend to cluster together. Before the initial confirmed COVID-19 cases in Chicago, the weighted clustering coefficient in Near North Side, Loop, Lincoln Park (community code 7), and Near West Side are higher than in other community areas in Chicago. It is interesting to note that these community areas are adjacent to each other, implying that there are more taxi drivers in these areas than in other community areas. The taxi drivers and the commuters are thus clustered within these four areas of Chicago.

## 5 Conclusion and discussion

Our exploratory analysis using network metrics captures interesting insights on how the COVID-19 pandemic affected taxi trip patterns in Chicago. We observe a rapid decrease in mobility during the initial outbreak of the pandemic in March 2020. However, from April 2020, we also observe a recovery of the transportation activity in Chicago. Even though there was an increase in mobility, it did not attain the pre-pandemic level and stabilized from July 2020 and continues to be around the same level. This indicates that the individuals may have reduced non-essential leisure and entertainment-related travel and may have adapted to working from home settings. Only those whose job structures may not allow setting up home offices might contribute to current mobility levels. Further, the stabilized mobility level after July 2020 could be attributed to trips related to unavoidable household-related necessities [55].

Further, we observe a heterogeneous impact of disease spread on mobility in various community areas. We find that there was a sharp decline in travel-related demand during the onset of the pandemic in regions with high economic activities in the pre-COVID-19 period (such as airports, downtown areas, and business zones). However, the regions characterized by low travel-related demand were not impacted much by the spread of the disease. The above observation indicates that COVID-19 drastically affected the individuals’ tendency to travel for work-related and leisure-related purposes.

Next, we find that disease outbreak impacts the flow of traffic in the city. In March 2020, COVID-19 cases started appearing in different regions in the city, due to which individuals tend to change their travel routes, which further impacts the traffic congestion in various areas. Therefore, during the initial spread of the disease, social planners must carefully monitor traffic movements at various locations in the city. Further, they need to ensure that all the disease prevention measures such as social distancing are carefully monitored not only in the high congestion areas during the pre-pandemic period but also in new congested regions that may emerge due to changes in travel patterns during the onset of the pandemic. After the overall mobility is stabilized, city planners may continue to carefully monitor a few highly congested regions as individuals have adapted to new travel patterns.

Finally, our analysis reveals a steady increase in short-distance mobility to nearby connected areas compared to long-distance travel during the onset of a pandemic. However, during the initial recovery period, there is again an increase in long-distance travel. During the later phase of the pandemic, the mobility network stabilizes with more clustered trips to nearby areas than less clustered long-distance trips, which were observed during the pre-COVID-19 period. This indicates that during the ongoing crisis, individuals tend to travel mostly to nearby locations for unavoidable necessities like shopping for household essentials. Based on the above insight, it is also crucial for social planners to identify clusters/communities of various mobility networks. The community area with a high local clustering coefficient plays an important role in propagating infection during the pandemic. Therefore, the identification of such high clustering zones is vital to limit the spread of COVID-19.

## Supporting information

S1 Fig Map of Chicago along with the taxi zones. (TIF)

S1 Table Community area codes and the names of the corresponding community areas. (DOCX)
