# Supplementary figures and images for: Impact of COVID-19 on the mobility patterns: An investigation of taxi trips in Chicago
Source: PLoS One. 2022 May 5;17(5):e0267436. doi: 10.1371/journal.pone.0267436 (PMC9070917; doi:10.1371/journal.pone.0267436)

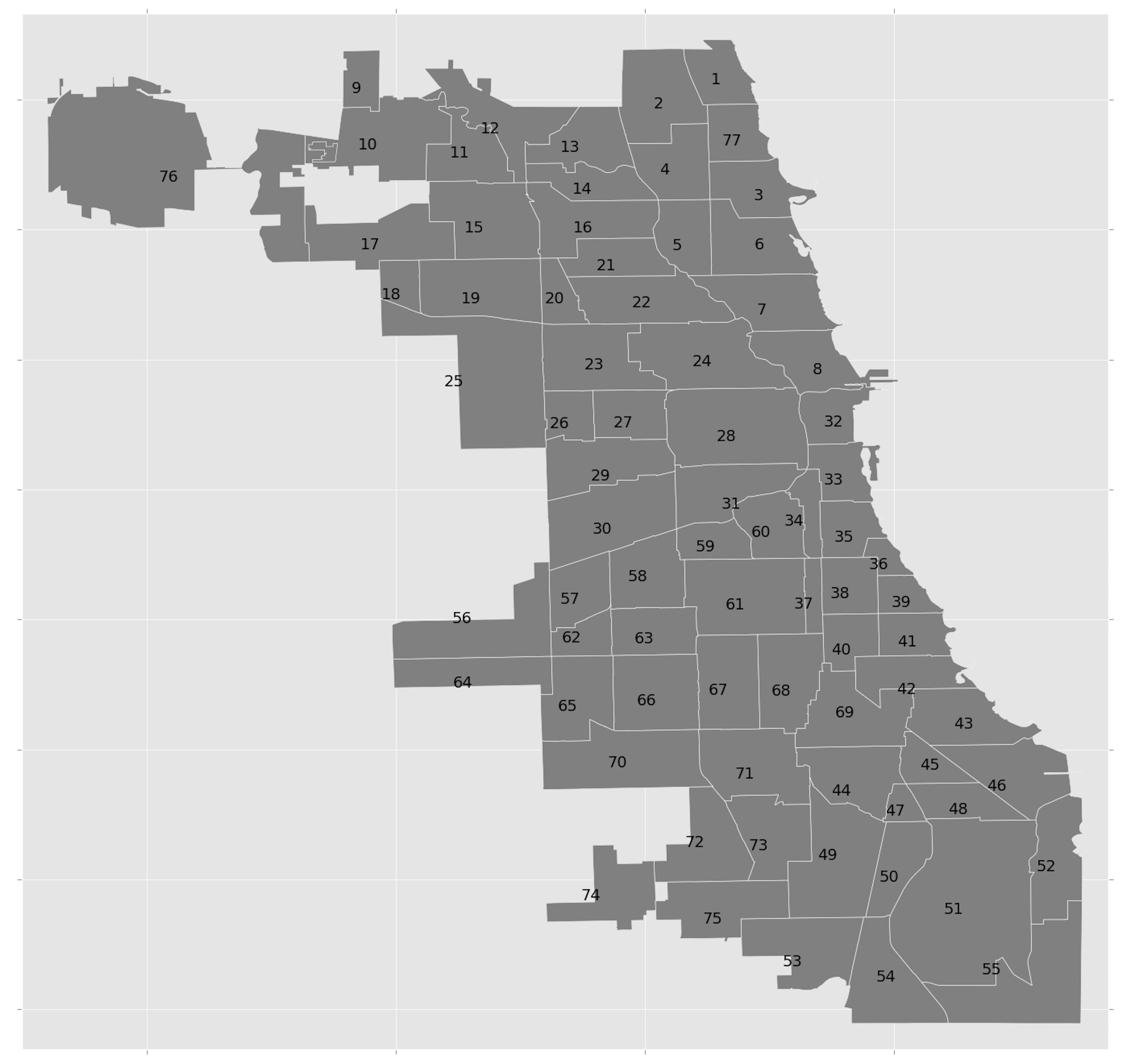

Supplement: S1 Fig — (TIF) [file pone.0267436.s001.tif]
