# Supplementary material for: Impact of COVID-19 on the mobility patterns: An investigation of taxi trips in Chicago
Source: PLoS One. 2022 May 5;17(5):e0267436. doi: 10.1371/journal.pone.0267436 (PMC9070917; doi:10.1371/journal.pone.0267436)
Supplement: S1 Table — (DOCX) [file pone.0267436.s002.docx]

**Supporting Information to *Impact of COVID-19 on the mobility patterns: An investigation of taxi trips in Chicago***

| **Code** | **Name** | **Code** | **Name** | **Code** | **Name** |
| --- | --- | --- | --- | --- | --- |
| 1 | Rogers Park | 26 | West Garfield Park | 51 | South Deering |
| 2 | West Ridge | 27 | East Garfield Park | 52 | East Side |
| 3 | Uptown | 28 | Near West Side | 53 | West Pullman |
| 4 | Lincoln Square | 29 | North Lawndale | 54 | Riverdale |
| 5 | North Center | 30 | South Lawndale | 55 | Hegewisch |
| 6 | Lake View | 31 | Lower West Side | 56 | Garfield Ridge |
| 7 | Lincoln Park | 32 | Loop | 57 | Archer Heights |
| 8 | Near North Side | 33 | Near South Side | 58 | Brighton Park |
| 9 | Edison Park | 34 | Armour Square | 59 | McKinley Park |
| 10 | Norwood Park | 35 | Douglas | 60 | Bridgeport |
| 11 | Jefferson Park | 36 | Oakland | 61 | New City |
| 12 | Forest Glen | 37 | Fuller Park | 62 | West Elsdon |
| 13 | North Park | 38 | Grand Boulevard | 63 | Gage Park |
| 14 | Albany Park | 39 | Kenwood | 64 | Clearing |
| 15 | Portage Park | 40 | Washington Park | 65 | West Lawn |
| 16 | Irving Park | 41 | Hyde Park | 66 | Chicago Lawn |
| 17 | Dunning | 42 | Woodlawn | 67 | West Englewood |
| 18 | Montclaire | 43 | South Shore | 68 | Englewood |
| 19 | Belmont Cragin | 44 | Chatham | 69 | Greater Grand Crossing |
| 20 | Hermosa | 45 | Avalon Park | 70 | Ashburn |
| 21 | Avondale | 46 | South Chicago | 71 | Auburn Gresham |
| 22 | Logan Square | 47 | Burnside | 72 | Beverly |
| 23 | Humboldt park | 48 | Calumet Heights | 73 | Washington Height |
| 24 | West Town | 49 | Roseland | 74 | Mount Greenwood |
| 25 | Austin | 50 | Pullman | 75 | Morgan Park |
|  |  |  |  | 76 | O'Hare |
|  |  |  |  | 77 | Edgewater |

**S1 Table. Community area codes and the names of the corresponding community areas.**
